# The Influence of Parasite Infections on Host Immunity to Co-infection With Other Pathogens

**DOI:** 10.3389/fimmu.2018.02579

**Published:** 2018-11-08

**Authors:** Neil A. Mabbott

**Affiliations:** The Roslin Institute & Royal (Dick) School of Veterinary Studies, University of Edinburgh, Edinburgh, United Kingdom

**Keywords:** parasite, helminth, co-infection, pathogenesis, susceptibility, immunity

## Abstract

Parasites have evolved a wide range of mechanisms that they use to evade or manipulate the host's immune response and establish infection. The majority of the *in vivo* studies that have investigated these host-parasite interactions have been undertaken in experimental animals, especially rodents, which were housed and maintained to a high microbiological status. However, in the field situation it is increasingly apparent that pathogen co-infections within the same host are a common occurrence. For example, chronic infection with pathogens including malarial parasites, soil-transmitted helminths, *Mycobacterium tuberculosis* and viruses such as HIV may affect a third of the human population of some developing countries. Increasing evidence shows that co-infection with these pathogens may alter susceptibility to other important pathogens, and/or influence vaccine efficacy through their effects on host immune responsiveness. Co-infection with certain pathogens may also hinder accurate disease diagnosis. This review summarizes our current understanding of how the host's immune response to infection with different types of parasites can influence susceptibility to infection with other pathogenic microorganisms. A greater understanding of how infectious disease susceptibility and pathogenesis can be influenced by parasite co-infections will enhance disease diagnosis and the design of novel vaccines or therapeutics to more effectively control the spread of infectious diseases.

## Introduction

The important host-parasite interactions that influence the progression and control of infection to individual pathogenic microorganisms have been studied in much detail, especially in laboratory mice. However, since many pathogens are acquired through similar routes of exposure (e.g., orally following ingestion of contaminated water, food, or pasture) infection of natural host species in field situations and humans with multiple pathogens is common, especially in regions with poor sanitation and/or limited access to clean drinking water. In order to sustain chronic infections, the parasitic microorgansisms have evolved a diverse range of mechanisms to enable them to evade or manipulate the host's immune response. As a consequence, by-stander effects due to infection with certain parasite species can significantly alter susceptibility to other important pathogens, and/or influence the development of pathology. Discussed throughout this review are examples of the many studies that have investigated the effects of parasitic helminth infections on host susceptibility to co-infection with a diverse range other pathogenic microorganisms.

Helminths are large, multicellular parasitic microorganisms. Estimates suggest that approximately a third of the global human population may be infected with helminth parasites, causing important public health concerns, especially in regions with poor sanitation or limited access to clean drinking water. Helminths are commonly referred to as parasitic worms and can include the following groupings: roundworms (nematodes), such as *Ascaris lumbricoides*; whipworms, such as *Trichuris trichiura* in humans and *T. muris* in mice; hookworms, such as *Necator americanus*; flukes (trematodes), such as *Facsiola hepatica* and *Schistosoma mansoni*; and cestodes (tapeworms). Many of these helminth parasites are soil-transmitted and cause gastrointestinal infections following ingestion of pasture or water contaminated with their eggs. The schistosomes, in contrast, can establish chronic infection within the host's bloodstream. Infections with helminth parasites often cause significant pathology, for example as they migrate through host tissues following infection via the skin (e.g., schistosomes), or feed on the gut epithelium (e.g., *Trichuris* spp.) (1). These chronic infections are often associated with the development of systemic and mucosal CD4^+^ T helper cell type 2 (Th2) polarized immune responses. These are typically characterized by increased expression of cytokines such as interleukin-4 (IL-4), IL-13, eosinophilia, production of immunoglobulin E (IgE), and stimulation of alternatively activated (M2) macrophages and type 2 innate lymphoid cells (ILC2) (2, 3). The alternatively activated macrophages are considered to play an important role in repairing the tissue damage caused by the helminth infection.

However, the characteristics of the immune response to infection with other types of parasites can differ substantially. Trypanosomes and malaria parasites are important vector-borne unicellular protozoan parasites, and are the causative agents of trypanosomiasis and malaria, respectively, in humans and animals. Each of these parasite species can establish chronic infections in the host's bloodstream: the trypanosomes living extracellularly, whereas the malaria parasites establish cyclical rounds of intracellular infection within erythrocytes. Host immune responses to infection with these protozoan parasites are usually fundamentally distinct from the predominantly Th2-polarized responses induced by helminth infections. Malaria infections, for example, are associated with elevated levels of pro-inflammatory cytokines such as interferon-γ (IFNγ), IL-12, increased levels of CD4^+^ Th1 cells, CD8^+^ T cells, and NK cells and the stimulation of pro-inflammatory classically activated (M1) macrophages (4, 5).

An increasing body of data indicates that alterations to these responses due to co-infection with other pathogens can dramatically influence disease susceptibility. Many of the examples presented below suggest links between parasite-induced disturbances to the Th1/Th2 balance and/or macrophage phenotype (alternatively activated vs. classically activated) and altered susceptibility or pathogenesis following subsequent co-infection with other pathogens. The effects of the parasite infection on the host's immune response may also have serious implications for the accurate diagnosis of other infections within the same individual. Bovine tuberculosis is an important infectious disease of cattle caused by the pathogenic bacterium *Mycobacterium bovis*. Current measures to control this disease include the regular testing of cattle and removal of infected stock from herds. A widely used diagnostic test for this disease is based on the detection of *M. bovis*-specific IFNγ production by peripheral blood lymphocytes. However, as helminth infections can modulate host IFNγ responses, co-infections with these parasites could have serious consequences for the reliable detection of other important pathogens (see section *Mycobacterium tuberculosis*).

This review therefore discusses how the induction of an immune response to infection with distinct types of parasites may modulate the development of effective immunity and/or disease pathogenesis to co-infections with other important pathogenic microorganisms. Many of the data obtained in the studies described below was derived from experimental mouse models, but studies from humans and natural host species are included where data were available.

## Malaria

Malaria, caused by infection with unicellular protozoan *Plasmodium* parasites, is an important infectious disease of humans and animals that inflicts significant morbidity and mortality in tropical and sub-tropical regions throughout the world. Infections are transmitted through the deposition of sporozoite parasite stages into the skin via the bite of infected mosquito vectors. These then travel via the bloodstream to the liver, where they infect hepatocytes and undergo morphological change and replication before subsequently infecting erythrocytes. The parasites then undergo extensive cycles of replication and infection in erythrocytes leading to anemia. During the erythrocytic stage of malaria infection, IFNγ production from CD4^+^ Th1-cells and CD4^+^ T-cell help for the B-cell response are each required for effective control and elimination of the parasitaemia. The actions of CD4^+^ T cells are also important for controlling the pre-erythrocytic stages of infection through the activation of parasite-specific CD8^+^ T cells [for in-depth review see (5)]. However, excessive inflammatory responses in response to malaria infection can also lead to significant immunopathology.

### Effects of malaria on helminth co-infection

Malaria patients in endemic regions are often co-infected with soil-transmitted helminths. Following infection of the mammalian host the larval stages of certain parasitic helminth species migrate to the lungs of the host where they cause pathology. The larvae typically induce strong pulmonary Th2 immune responses and are accompanied by the induction of alternatively activated macrophages. The combined actions of these immune responses are considered to help mediate parasite clearance and the repair of host tissues. In BALB/c mice infected with hookworms parasites such as *Nippostrongylus brasiliensis*, the development of a Th2-polarized immune response to the hookworm infection was impaired in those co-infected with *P. chabaudi chabaudi* malaria parasites. This effect also coincided with the reduced expression of alternatively activated macrophage-derived factors in the lung such as chitinase and resistin family members (6). Similarly, in a separate study the incidence of lung granulomas induced by infection with *Litomosoides sigmodontis* microfilariae was reduced in mice co-infected with either *P. chabaudi* or *P. yoelii* malaria parasites (7). These experimental mouse studies suggest that malaria infection can negatively impact on the ability of the host to induce a Th2-polarized specific immune response to co-infection with helminths. How malaria infection mediates these effects is not known. Further experiments are clearly necessary to determine whether they are a consequence of direct effects of the malaria parasites themselves or are an indirect consequence of the induction of a strong Th1-polarized immune response/cytokine milieu in response to the malaria infection.

### Effects of helminth co-infection on malaria

Co-infections with parasites such as helminths may also have a significant impact on malaria pathogenesis and susceptibility (8). In mice infected with *N. brasiliensis* 2 weeks before subsequent infection with *P. berghei* malaria parasites, the induction of a malaria-driven Th1 cytokine response was impeded, affecting the activation phenotype of the macrophages in the lung (8). Helminth infections can promote the secretion of IL-10 by regulatory T cells (9, 10), and this can downregulate the expression of pro-inflammatory cytokines such as IL-12p70 and IFNγ. Since IFNγ plays an important role in protective immunity to *P. falciparum* infection, it is plausible that expression of an anti-inflammatory cytokine milieu during helminth infection might exacerbate malaria pathogenesis and susceptibility. In support of this hypothesis, co-infection of mice with the hookworm parasite *N. brasiliensis* was reported to impede the induction of a pro-inflammatory classically activated macrophage phenotype in the lungs of mice subsequently infected with malaria parasites (8). The induction of an effective Th1 response to *P. berghei* infection was similarly reduced in mice co-infected with schistosomes (*S. mansoni*). As a consequence, the co-infected mice were less able to control the malaria infection, displaying increased parasitemias during the early phase of *P. berghei* infection and increased fatality (11). Mice chronically infected with the gastrointestinal helminth pathogen *Heligmosomoides polygyrus* before a subsequent *P. chabaudi* AS infection likewise displayed reduced immunity to the bloodstream stage of malaria infection, including decreased production of IFNγ and malaria-specific Th1-associated IgG2a antibody production (12).

Parasite co-infections may also affect the magnitude of the pathology that develops in host tissues. For example, an exacerbation of immunopathology during *P. chabaudi* infection was observed in mice co-infected with *L. sigmodontis* microfilariae (13). The incidence of pathology in the co-infected mice was found to be associated with the absence of microfilaraemia. This implies that the presence of the microfilariae stimulates an immunomodulatory response in the host that can partially protect against severe malaria (13). However, helminth co-infections may differentially regulate murine malarial infections in a malaria strain-dependent way. For example, co-infection of mice with *L. sigmodontis* impaired the development of lesions in the kidneys caused by infection with either *P. chabaudi* or *P. yoelii*. However, the peak of malaria parasitaemia was decreased in mice co-infected *P. yoelii*, but not in those co-infected with *P. chabaudi* (7). Conversely, the growth of *P. yoelii* in the liver was inhibited in mice co-infected with *S. mansoni*, and malaria parasite gametocyte infectivity was much reduced (14).

Whether co-infections with helminths such as schistosomes can modulate susceptibility to malaria in humans is uncertain, but an experimental study in baboons (*Papio anubis*) suggests that this also plausible. This study showed that the baboons that were chronically infected with *S. mansoni* had significantly lower *P. knowlesi* malaria parasite burdens and were protected from anemia (15). A study of Senegalese children similarly showed that those with light *S. haematobium* infection had lower burdens of *P. falciparum* when compared to those that weren't co-infected with schistosomes (16). The mechanisms responsible for mediating these effects are not known. It is possible that the reduced levels of regulatory T cells or a Th2-polarized cytokine milieu in *S. haematobium* infected children may provide protection against falciparum malaria by modulating systemic expression of Th1 cytokines (17).

### Effects of parasite co-infection on experimental cerebral malaria

Severe forms of malaria are leading causes of mortality in infected children and pregnant mothers, presenting as cerebral malaria, severe anemia, or acidosis (18). The sequestration of malaria parasites across the blood-brain barrier leads to the influx of immune lymphocytes and leukocytes into the brain, particularly parasite-specific CD8^+^ T cells, which ultimately leads to the development of neuropathology. Studies using a murine experimental cerebral malaria (ECM) model show that co-infection with Chikungunya virus can impede the sequestration of malaria parasites into the brain and provide some protection against ECM (19). In this model co-infection system, the malaria parasite-specific pathogenic CD8^+^ T cells appeared to be retained in the spleens of mice co-infected with Chikungunya virus due to their reduced expression of the CXCR3 chemokine receptor (19). This implied that the reduced early migration of pathogenic CD8^+^ T cells into the brains of the virus co-infected mice may have impeded the development of ECM and brain pathology.

Systemic co-infection of mice with the schistosomes (*S. mansoni*) seven weeks before infection with *P. berghei* malaria parasites was similarly shown to reduce the severity of ECM (11, 20). In this study, protection from ECM was dependent on the relative doses of the parasites used in the co-infections. Here, infection with a high dose of schistosome cercariae resulted in higher protection against ECM when co-infected with a low dose of *P. berghei* malaria paratites. Conversely, when the mice were co-infected with *S. mansoni* and a higher dose of *P. berghei*, the development of ECM was unaffected when compared to mice infected with malaria parasites alone (21). Development of ECM in mice is considered to be Th1-associated. The switch toward a Th2-polarized cytokine profile in the *S. mansoni* co-infected mice suggests a mechanism by which the effects on ECM pathogenesis may have been mediated. Clearly further experiments are required to specifically confirm the identify of the cellular and molecular factors responsible for these effects. A predominantly Th1-polarized cytokine profile is observed in mice by 4 weeks after *S. mansoni* infection (before significant egg production occurs). When mice were co-infected with malaria parasites 4 weeks after *S. mansoni* infection (prior to the onset of the Th1/Th2 switch), no improvements to ECM pathogenesis were observed (20), supporting the hypothesis that a Th2-polarized cytokine response may have a protective role.

Co-infections with the helminth parasites *A. lumbricoides* and *T. trichiura* have also been associated with reductions in risk of cerebral malaria in humans (22). However, it is interesting to note that the beneficial effects of *T. trichiura* were reduced when the individuals were also co-infected with hookworms (22). This study reveals an additional layer of complexity in naturally affected host-species where the outcome on the host of interactions between two parasite species can be modified by the presence of a third. These data emphasize how the relationships between multiple parasite communities in an individual host can significantly affect disease outcomes.

## Trematodes (flukes)

### Liver fluke

The liver fluke *Fasciola hepatica* is highly prevalent in ruminant livestock species, but can also establish infection in a wide range of other mammalian species including humans. Infection of livestock with *F. hepatica* occurs through the ingestion of pasture contaminated with encysted cercariae. The parasites then migrate through the gut wall and peritoneal cavity to the liver and bile ducts, where they mature and produce large numbers of eggs that are excreted into the environment. In common with many helminth infections, the migrating juvenile parasite stages induce a strong Th2-polarized immune response in the mammalian host. Infection of mice with *F. hepatica* can down-regulate Th1-responses *in vivo*, even in IL-4-deficient mice (23) suggesting this is not simply due to the actions of Th2 cells or cytokines. Indeed, the parasites produce a large range of excretory/secretory (ES) molecules that help them to mediate tissue invasion, feeding and immunomodulation (24) including the suppression of IFNγ responses (25).

Bovine tuberculosis (BTB), caused by infection with the bacterium *M. bovis* is an important pathogen of cattle worldwide and has zoonotic potential. Although some countries have reduced or eliminated BTB, wildlife *M. bovis* reservoirs and the limitations of diagnostic tests have hindered successful eradication in other regions. The single intradermal comparative cervical tuberculin test (SICCT) and the *in vitro* IFNγ assay are commonly used to identify *M. bovis* infected cattle. However, certain parasite co-infections can reduce the sensitivity of these assays. A large-scale epidemiological study in England and Wales showed that in dairy herds with a high incidence of *F. hepatica* fewer animals were detected as positive reactors against BTB (26). Experiments in cattle confirmed that infection with *F. hepatica* reduces the sensitivity of the standard BTB tests (SICCT test and IFNγ test) through reduced *M. bovis*-specific Th1 immune responses (27). However, *F. hepatica* co-infection did not influence the detection of visible lesions in BTB infected cows (28). These data clearly show how the immunomodulatory effects of *F. hepatica* on the bovine host can reduce the sensitivity of current pre-mortem BTB tests. This may significantly influence the ability of practical measures to eliminate BTB infections in regions with high prevalence of *F. hepatica*.

Despite the reduced *M. bovis*-specific Th1 immunity in cows co-infected with *F. hepatica*, mycobacterial burdens were shown to be reduced (29). The mechanisms responsible for this are uncertain, but the authors proposed that the actions of alternatively-activated macrophages induced in response to *F. hepatica* infection may act to limit the proliferation of *M. bovis*.

In sheep, infections with the liver flukes *Dicrocoelium dentriticum* or *F. hepatica* can predispose the ewes to mastitis in the immediate *post-partum* period (30). Abnormal metabolism of carbohydrates during the later stages of pregnancy in ewes can cause pregnancy toxemia. This disease is associated with hyperketonaeimia, and blood concentrations of the ketone body β-hydroxybutyrate can help detect ewes at risk of developing pregnancy toxemia. Higher concentrations of β-hydroxybutyrate were recorded in trematode-infected ewes (30). Since β-hydroxybutyrate may have immunomodulatory effects, the authors hypothesized that its increased levels in infected ewes may suppress immunity in the udder enhancing the risk of infection at this site (30). This has raised the hypothesis that the use of anthelmintic drugs may also help reduce the incidence of mastitis in trematode-affected flocks.

Finally, infection with the human liver fluke *Opisthorchis viverrini* is endemic in the Greater Mekong sub-region. This fluke is classified as a group 1 carcinogen by the International Agency for Research on Cancer because chronic infections with *O. viverrini* can lead to the development of cholangiocarcinoma, a malignant cancer of the bile ducts. In areas of Thailand where flukes are endemic, the Gram-negative bacterium *Helicobacter pylori* was found in 66.7% of the patients studied that had cholangiocarcinoma (31). This raised the hypothesis that co-infections with *H. pylori* and with *O. viverrini* synergistically increases the severity of hepatobiliary abnormalities. Indeed, in an experimental hamster model the severity of the hepatobiliary abnormalities was increased in those that were co-infected with *O. viverrini* (32).

### Effects on schistosome (blood fluke) infections

Infections with schistosomes cause chronic inflammatory disease in humans and animals. The diseases they cause can lead to the development of severe pathology, significant morbidity, and economic loss. Schistosomes are transmitted through the skin via contact with infected water sources inhabited by the snail vector. The parasites establish chronic infections in the mammalian host's bloodstream where they mature, mate, and produce substantial quantities of eggs. The eggs enter the intestines and bladder where they are excreted via feces and urine. Deposition of the eggs in host tissues can cause chronic inflammation, tissue damage and fibrosis. Schistosomes typically induce a Th2-polarized immune response in the mammalian host that enables them to establish chronic infections where they can persist for years (33).

Experiments in mice have shown that a gastrointestinal nematode infection can influence the pathogenesis of a subsequent schistosome infection. A chronic *Trichuris muris* infection within the large intestine enhanced the survival and migration of *S. mansoni* schistosomula to the portal system. Consequently, schistosome worm and egg burdens and associated pathology were enhanced when compared to mice infected with *S. mansoni* alone (34). This suggests that the immunomodulatory effects elicited in the mucosa of the large intestine to enable the gastrointestinal helminth to establish chronic infection can extend to other host tissues. This may exacerbate host susceptibility to subsequent co-infection with other helminth parasites. A similar effect has been reported to occur in a naturally-affected livestock species. A longitudinal study of free-ranging African buffalo reported that animals infected with *Cooperia fuelleborni* had greater burdens of schistosomes (*Schistosoma mattheei*) than those where this nematode species was not detected (35). The mechanisms that mediate these effects in the co-infected animals remain to be determined. This could simply represent host variation in susceptibility to gastrointestinal nematodes. However, since infection of cattle with the related gastrointestinal nematode species *C. oncophora* enhanced their susceptibility to lungworm (*Dictyocaulus viviparus*) infection (36), it is plausible that nematode-mediated effects on the buffalo immune response may similarly impede the establishment of immunity to schistosomes.

However, it is important to note that data from mouse studies show that differences in the species of the co-infecting parasite may have contrasting effects on schistosome infections (34, 37). The gastrointestinal helminth *H. polygyrus* establishes infection specifically within the murine duodenum. In mice infected with *H. polygyrus* a marked reduction in schistosome egg-induced hepatic pathology was observed. This effect appeared to correlate with significant decreases in the expression of pro-inflammatory cytokines responsible for the induction of the egg-induced immunopathology (37). Thus, although co-infection with gastrointestinal helminths may significantly impact on schistosomiasis, the currently available data do not help us to reliably predict whether they are likely to exacerbate or reduce the disease pathogenesis.

## Trypanosomes

### African trypanosomes

African trypanosomes are single-cell extracellular hemoflagellate protozoan parasites and are transmitted between mammalian hosts via blood-feeding tsetse flies of the genus *Glossina*. The *Trypanosoma brucei rhodesiense* and *T. b. gambiense* subspecies cause human African trypanosomiasis in endemic regions within the tsetse fly belt across sub-Saharan Africa. Animal African trypanosomiasis is caused by *T. congolense, T. vivax*, and *T. brucei* and inflicts substantial economic strains on the African livestock industry. The parasitic life cycle within the mammalian host is initiated by the intradermal injection of metacyclic trypomastigotes by the tsetse fly vector. The extracellular parasites then reach the draining lymph nodes, presumably via invasion of the afferent lymphatics and then disseminate systemically (38, 39). During this process the metacyclic trypanosome forms differentiate into long slender bloodstream forms that are adapted for survival within the mammalian host. When C57BL/6 mice are infected with *T. brucei* the initial parasitaemic wave coincides with the expression of high levels of IFNγ by the host in an attempt to control the infection. However, the trypanosomes also cause significant immunosuppression enabling them to establish chronic infections in the hostile environment of the host's bloodstream.

A study has shown that the severity of malaria and trypanosomiasis was exacerbated in mice co-infected with *P. berghei* and *T. brucei* (40). In the co-infected mice survival rates were reduced and the parasitaemias were greater, with more severe anemia and hypoglycaemia. Each of these infections induces a strong pro-inflammatory response with high levels of IFNγ. Further studies are necessary to determine whether additive/synergistic effects of each infection on IFNγ expression are responsible for the increased disease severity observed in the co-infected mice.

Trypanosome infections may also modulate host susceptibility to infection with some pathogenic bacteria. For example, in mice chronically-infected with either *Brucella melitensis, B. abortus*, or *B. suis*, the burden of bacteria in the spleen was reduced if the mice were also co-infected with *T. brucei* (41). The effects of *T. brucei* infection on *Brucella* burdens in co-infected mice were impeded in the absence of functional IL-12p35/IFNγ signaling. This suggests that the strong pro-inflammatory IFNγ-mediated immune response induced by the *T. brucei* infection aided the clearance of *Brucella*. Thus, although infections with *T. brucei* can induce significant levels of immunosuppression and immunopathology, this study shows that under some circumstances the host's response to *T. brucei* infection may provide protection against co-infection with other pathogens (41). However, this not true for all pathogenic bacteria. Although Th1 responses are essential for protection against *Mycobacterium tuberculosis, T. brucei* infection did not ameliorate the susceptibility of mice to co-infection with this pathogenic bacterium (41).

### T. cruzi

Infection with the obligate intracellular parasite *T. cruzi* causes Chagas' disease and is an important cause of morbidity and mortality in humans in Central and South America (42). Concurrent infection of mice with *S. mansoni* can significantly affect disease pathogenesis and susceptibility following infection with *T. cruzi* (43). The co-infected mice were shown to be unable to effectively control the *T. cruzi* infection, and the increased parasitic load was accompanied by substantial inflammation in their livers. Infections with *T. cruzi* are associated with the establishment of a Th1-polarized immune response, and the production of macrophage-derived NO from arginine by inducible NO synthetase (iNOS) is important for parasite clearance. However, during the chronic phase of schistosomiasis high levels of arginase-1 are instead expressed by alternatively activated macrophages. In the *S. mansoni* co-infected mice, the reduced protection against *T. cruzi* coincided with the reduced production of IFN-γ and NO (43). This study illustrates how macrophage polarity and the relative expression levels of iNOS and arginase-1 in response to infection with one parasite could influence the host's ability to co-infection with other parasites.

## Apicomplexan parasites

### Toxoplasma gondii

*T. gondii* is an orally-acquired obligate intracellular protozoan parasite affecting ~30% of the human population. Following oral infection, the parasites typically disseminate systemically and convert into dormant stages in muscle tissues and the brain. Infection with *T. gondii* elicits a strong Th1-polarized immune response characterized by production of IFNγ, IL-12, and parasite specific IgG2a antibodies. Studies in mice suggest these responses are important for host protection. Infections are typically asymptomatic in immunocompetent humans, but can reactivate in immunocompromised individuals and result in the development of a life-threatening encephalitis.

Although a study has shown that gastrointestinal helminth (*H. polygyrus*) co-infection did not affect the induction of a protective Th1 response to *T. gondii* infection (44), the Th2-response to the *H. polygyrus* infection was impeded. Co-infection with *T. gondii* in these mice was instead accompanied by a shift toward a non-protective helminth-specific Th1 response (44). Conversely, separate studies have shown that *H. polygyrus* co-infection can negatively impact upon CD8^+^ T cell differentiation and cytokine production in response to *T. gondii* infection (45, 46). Conventional dendritic cells from the *H. polygyrus* co-infected mice also had reduced expression of IL-12 (46). The reasons for the contrasting outcomes in these two studies are not immediately apparent, but are perhaps explained by important differences in their experiment design. In the first study (44), the mice were first infected with *T. gondii* and 14 days later orally co-infected with *H. polygyrus*. However, in the other studies (45, 46), the mice were first infected with *H. polygyrus* and subsequently co-infected with *T. gondii* parasites.

Although further experiments are necessary to confirm the cellular and molecular mechanisms that underpin these effects, these studies suggest that differences in the order and timing of the infections with two distinct oral pathogens, can significantly influence the host's response to the second. In particular these data imply that the presence of a strong Th1-polarized immune response to an ongoing *T. gondii* infection can impede the development of Th2-polarized immune responses to a subsequent helminth co-infection (44). However, the induction of CD8+ T-cell responses against *T. gondii* co-infection is impeded in the presence of an existing Th2-polarized immune response to a gastrointestinal helminth infection.

### Eimeria

*Eimeria tenella* is a major pathogen of chickens causing intestinal coccidiosis. Infection with *E. tenella* is restricted to the caecum and causes significant morbidity and mortality, including diarrhea, mucosal lesions and weight loss. The prevalence of *E. tenella* and *T. gondii* is widespread, suggesting co-infections with these two pathogens may be common. The effect of co-infection of chickens with *Eimeria* and *T. gondii* has been addressed in an experimental study (47). However, the pathology and immune response following co-infection with these parasites did not differ significantly from that observed in chickens infected with *Eimeria* alone. Co-infection with *Eimeria* also did not affect the abundance of *T. gondii*-positive tissue samples or the clinical course of *T. gondii* infection. Distinct *T. gondii* strain types have been characterized in Europe and North America (type I, II, and III) and these can significantly influence the virulence and severity of the disease in different host species (48). For example, chickens are generally considered refractory to infection with type I or II oocysts (49, 50), but they may cause severe disease in other host species such as mice (51). Whereas, co-infection of chickens with two common apicomplexan parasites did not reveal any significant mutual effects on disease pathogenesis (47), a study of wild rabbits in Scotland suggested a link between *T. gondii* infection and higher burdens of *E. stiedae* (52). Whether differences in the virulence of *T. gondii* infection in different host species could have a significant impact on their susceptibility to co-infection with other pathogens remains to be determined. Of course, the possibility also cannot be excluded that the rabbits with high *Eimeria* burdens had high susceptibility to orally-acquired parasite infections.

### Giardia

Infection with *Giardia duodenalis* (syn. *G. intestinalis*, or *G. lamblia*) is a leading cause of waterborne diarrhoeal disease. The characteristic signs of Giardiasis include diarrhea, abdominal pain, nausea, vomiting, and anorexia, with some individuals also developing extra-intestinal and post-infectious complications. Little is currently known of the effects of parasite co-infections on susceptibility to giardiasis. A study of 3 year old children in the Quininde district of Ecuador has shown that those co-infected with the gastrointestinal helminth *A. lumbricoides* had plasma cytokine profiles indicative of an increased Th2/Th1 cytokine bias, and significantly lower plasma levels of IL-2 and TNF-α when compared to those infected with *G. lamblia* alone (53). Studies from mouse models have shown that expression of TNF-α is essential for resistance to *G. lamblia* infection (54). Little is known of the mechanisms that are essential for protection of humans against *Giardia* infections. However, it is plausible that the effects of *Ascaris* infection on TNF-α expression may have negatively affected the ability of the children to eradicate *Giardia*.

## Effects on bacterial infections

### Effects on *Salmonella* pathogenesis

Oral infections with the Gram negative bacterium *Salmonella*, through consumption of contaminated food such as meat, eggs, or milk are amongst the most common causes of diarrhea. Co-infections with distinct parasites, helminths, and malaria parasites, have each been shown to exacerbate susceptibility to, or the pathogenesis of, salmonellosis.

Many of the examples discussed above have suggested a link between the induction of a Th2-polarized immune response to helminth infection and the reduced development of Th1-polarized immunity to co-infection with other pathogens. However, co-infection of mice with the gastrointestinal helminth *H. polygyrus* has also been shown to enhance the pathogenesis of infection with *Salmonella enterica* serovar Typhimurium independently of the actions of Th2 cells and regulatory T cells (55). Here, co-infection with *H. polygyrus* has been reported to disrupt the metabolic profile within the small intestine, and by doing so, directly affect the invasive capacity of *S. typhimurium*. This helminth infection was shown to mediate this effect through the enhancement of bacterial expression of *Salmonella* pathogenicity island 1 (SPI-1) genes (55). This study reveals a novel immune system-independent mechanism by which a helminth-modified metabolome in the host's intestine can promote susceptibility to bacterial co-infection.

Invasive nontyphoid *Salmonella* (NTS) bacteraemia is a common cause of community-acquired bacteraemia in the human populations of several regions of sub-Saharan Africa (56). Associations between NTS co-infection and high malaria mortality have been reported (57), and a study of hospitalized children in north-eastern Tanzania showed that a decline in malaria cases was associated with a similar decline in the incidence of NTS and other forms of bacteraemia (58).

Since malaria parasites establish infection within erythrocytes, the daily rounds of parasite replication within these cells can cause high levels of erythrocyte lysis and haemolysis, resulting in anemia. The association of NTS infection with haemolysis is well-established in humans with malaria, especially in patients with severe malarial anemia (59). This haemolysis releases large quantities of cell-free haeme which is toxic to the host. To counter this cytotoxicity, haeme oxygenase 1 (HO-1) expression is induced to degrade the haeme and provide tolerance toward some of the pathological consequences of malaria infection. The actions of HO-1 also provide an additional cytoprotective role by limiting the production of reactive oxygen species (60). However, data from mice (61) and from the analysis of neutrophils from malaria-infected children (62) show that the actions of HO-1 in granulocytes in response to haemolysis during malaria infection impedes their oxidative burst activity and production of reactive oxygen species. This leads to dysfunctional granulocyte mobilization and long-term neutrophil dysfunction. As a consequence, *Salmonella* are able to survive and proliferate within neutrophils during malaria infection due to their decreased oxidative burst activity, leading to increased NTS susceptibility (61, 62). These data clearly show how a host-induced cytoprotective response to one of the pathological consequences of malaria infection (haemolysis) can significantly impair neutrophil-mediated resistance to co-infection with another pathogen.

### Mycobacterium tuberculosis

Infection with the obligate intracellular bacterium *M. tuberculosis* causes tuberculosis (TB), a chronic disease affecting ~2 billion people worldwide. This infection typically affects the lungs, and the majority of individuals infected with *M. tuberculosis* have latent infections and are asymptomatic. However, data from various clinical studies in humans have raised the hypothesis that helminth co-infections may affect TB susceptibility and the risk of developing latent *M. tuberculosis* infection, as co-infected patients often displayed more advanced disease (63, 64). Gastrointestinal helminth infections may also modulate susceptibility and/or disease pathogenesis to co-infection with other pathogenic *Mycobacteria* spp. Infection with *M. leprae* or *M. lepromatis* causes leprosy, a chronic granulomatous infectious disease. A study of Indonesian patients infected with *M. lepromatis* reported that those with gastrointestinal helminth infections (e.g., *T. trichiura, Stongyloides sterocalis*) similarly presented with more severe types of leprosy (65).

Studies from animal models, especially mice, indicate that protection against TB infection is dependent upon the induction of a strong pro-inflammatory Th1-polarized immune response and production of IFNγ, IL-12, and TNF-α, as well as contribution from Th17 cells and IL-17 and IL-23 (66, 67). The immune response induced by co-infection with gastrointestinal helminths may affect immunity to *M. tuberculosis* through a range of distinct mechanisms, including the induction of regulatory T cell responses (68), modulation of Th1 and Th17 responses to the bacterial infection and reduced expression of effector cytokines (69–71). The increased expression of Th2 cytokines, especially IL-4, in mice co-infected with helminths also promotes the induction of alternatively activated macrophages. These cells have been shown to be less effective than pro-inflammatory IFNγ-stimulated macrophages in controlling *M. tuberculosis* (72, 73). Indeed, antigens from helminths such as *T. muris* and *Hymenolepis diminuta* (tapeworms) can induce an alternatively activated phenotype in human macrophages, reducing their ability to control *M. tuberculosis* infection *in vitro* (74). However, mouse studies show that under some circumstances the acute host response to helminth infection might enhance the early control of *M. tuberculosis* infection by alveolar macrophages (75).

Although the induction of T cell responses is considered essential for protective immunity against TB infection, B cells, and the production of mycobacterial-specific antibodies have also been proposed to play an important role (76). However, helminth infections can also influence B cell responses to *M. tuberculosis* infection. Co-infection of humans with *Strongyloides stercoralis* has been shown to affect B cell responses during latent tuberculosis infection, significantly reducing B cell numbers, the induction of mycobacterial-specific IgM and IgG resopnses and expression levels of the B-cell growth factors APRIL and BAFF (77). These helminth-associated impairments to mycobacteria-specific B cell responses could have significant implications for the efficacy of vaccine-induced immune responses to TB in affected regions. However, a clinical trial study in healthy, previously BCG vaccinated adolescents reported that helminth co-infection (*S. mansoni* did not impact on the efficacy of a candidate viral vector-based TB vaccine (78).

### Pneumonia

A study of goats in Nigeria revealed that the incidence of pneumonia corresponded with the presence of gastrointestinal parasitism in the same animals (79). Furthermore, a strong association was observed between the occurrence of helminth infections and granulomatous pneumonia. In this study the affected goats were often hydrated, implying that the gastrointestinal helminth infections may have caused pulmonary oedema due to increased fluid accumulation in the lung. The authors suggested that may this have reduced the efficacy of immunity in the lung, enabling other pathogenic microorganisms (bacteria and/or viruses) to establish infection and the subsequent development of pneumonia (79).

### Pathogenic *Escherichia coli*

The zoonotic bacterium *E. coli* O157 is a worldwide problem for public health causing haemorrhagic diarrhea in infected humans. Cattle are considered the major reservoir for human infection, and infection in these animals is usually asymptomatic. A study of 14 British farms suggested that co-infection with the liver fluke *F. hepatica* may increase the risk of *E. coli* O157 shedding (80). This implies that strategies aimed at controlling *F. hepatica* infection may have additional benefit by reducing the shedding of *E. coli* O157.

*Giardia lamblia* and enteroaggregative *E. coli* are two of the most commonly isolated pathogens in malnourished children. Mice fed on a protein-deficient diet were used to model the pathogenesis of co-infection with these pathogens in malnourished individuals (81). The malnourished mice fed a protein-deficient diet exhibited significantly greater weight loss following co-infection with *G. lamblia* and enteroaggregative *E. coli* when compared to co-infected mice that received a normal diet. This study reveals how the combined effects of the composition of the host's diet and pathogen infection can affect disease pathogenicity. Studies using a laboratory biofilm system to mimic the human gut microbiota have revealed that *G. duodenalis* can cause significant dysbiosis. These effects were mediated in part through the actions of secretory-excretory *Giardia* cysteine proteases, and these could promote gut epithelial cell apoptosis, tight junction disruption, and bacterial translocation across the gut epithelium (82).

Despite the ability of *Giardia* infection to cause intestinal dysbiosis, in countries with poor standards of sanitation *Giardia* infection has been associated with decreased incidence of diarrhoeal disease. This raised the hypothesis that infection with *Giardia* spp. might modulate host responses to co-infection with attaching and effacing enteropathogens. Weight loss, pathological signs of colitis and bacterial colonization and translocation were significantly attenuated in mice co-infected with *G. muris* and the attaching and effacing enteropathogen *Citrobacter rodentium* (83). These effects coincided with enhanced secretion of the antimicrobial factors β-defensin 2 and trefoil factor 3 by gut epithelial cells during co-infection (83). This suggests that components of the host response to infection with *Giardia* spp. (e.g., production of antimicrobial factors) may reduce susceptibility to gastrointestinal co-infection with certain pathogenic bacteria. These studies also highlight how differences in the anatomical niches that the parasites inhabit may also have a significant influence on the disease pathogenesis of a bacterial co-infection. When pathogens infect the same niche such as the gastrointestinal tract, infection with *Giardia* spp. may provide protection against bacterial co-infection (83). Conversely, infection with *F. hepatica* in the liver was associated with the enhanced pathogenesis of a bacterial co-infection in the intestine (80).

## Effects on viral infections

Several studies have revealed how parasite co-infections, especially helminths, can reduce immunity to important viral pathogens. In many of these instances the induction of a Th2-polarized immune response to the parasite infection appears to impede the development of effective antiviral immunity. For example, mice co-infected with the gastrointestinal helminths *Trichinella spiralis* or *H. polygyrus* and mouse norovirus (MNV) had increased viral loads and reduced levels of virus-specific CD4+ T cells expressing IFNγ and TNF-α when compared to mice infected with norovirus alone (84). The production of Th2 cytokines during helminth infection is associated with the expression of the transcription factor signal transducer and activator of transcription 6 (STAT6) by alternatively activated macrophages. In mice deficient in STAT6, viral loads were reduced when compared to wild-type controls indicating that the induction of STAT6-dependent alternatively activated macrophages during helminth infection can impede the induction of antiviral innate and adaptive immunity (85). As well as impeding the efficacy of anti-viral immunity, the expression of IL-4 and activation of the transcription factor STAT6 during helminth infection in mice can also promote the reactivation of a latent γ-herpesvirus infection (85). The sections below describe how parasite co-infections may impede immunity to certain viral pathogens, but examples are also provided where potential host-protective effects have been proposed.

### Impeding viral immunity

#### Hepatitis C virus (HCV)

Infections with HCV can cause liver fibrosis and cirrhosis and are major causes of chronic liver disease throughout the world. A study of Egyptian HCV patients co-infected with *S. mansoni* showed that these individuals had significantly higher concentrations of HCV proteins in distinct stages of the virus-mediated hepatic fibrosis (86). Co-infected individuals may also have an increased rate of progressing through the different pathological stages of HCV-mediated hepatic fibrosis than those infected with HCV alone. The authors suggested that the immune response induced in response to *S. mansoni* infection may have led to enhanced HCV propagation and increased concentration of HCV proteins. This implies that that components of the immune response to the schistosome infection may have suppressed the HCV-induced Th1 cytokine production, reducing antiviral immunity. Treatment of co-infected patients with anti-schistosome therapy may therefore help to decrease the progression rate of the HCV-induced hepatic fibrosis.

#### Human immunodeficiency virus (HIV)

African adults infected with HIV are often co-infected with gastrointestinal parasites. A study of HIV-infected Ugandans showed a high prevalence of parasitic infections (especially *Necator americanus*), and co-infection with hookworms correlated with much lower peripheral blood CD4^+^ T cell levels than those infected with HIV alone (87). This raises the suggestion that individuals co-infected with hookworms and HIV are at a distinct immunologic disadvantage when compared to those infected with HIV alone. This hypothesis has been experimentally tested in mice using a helminth/retrovirus co-infection model (88). Although the ability of the mice to control the *L. sigmodontis* infection was not affected in the co-infected mice, helminth infection did interfere with the host's ability to the control of the viral infection. Levels of virus-specific CD8^+^ T cells, FoxP3^+^ regulatory T cells, and cytokines were similar in co-infected mice and those infected with Friend virus alone. The increased viral loads in co-infected mice were instead associated with reduced titres of neutralizing virus-specific IgG2b and IgG2c antibodies. However, earlier studies in humans have reported no beneficial effect of antihelminthic treatment on HIV viral loads [plasma HIV-1 RNA concentrations; (89)], and other studies have suggested that helminth co-infections do not exacerbate HIV infection (90). On face value, the prospective studies undertaken in humans suggest that antihelmitic treatments or helminth-specific vaccines are unlikely to have any beneficial effects in regions with high incidence of helminth and HIV infections. However, further studies are necessary to determine whether additional factors such as host age, the intensity of the helminth infection or magnitude of the viral load affect the efficacy of such approaches.

#### Human T-cell lymphotropic virus-1 (HTLV-1)

*Strongyloides stercoralis* is a soil-transmitted intestinal helminth parasite of humans. Infection occurs following penetration of the skin by filiform larvae. The majority of *S. stercoralis* infections are asymptomatic to mild, but a life-threatening hyper-infection syndrome can develop in immunosuppressed hosts. This is accompanied by the massive dissemination of the filariform larvae from the colon to the lungs, liver, central nervous system, or kidneys. Co-infection with HTLV-1 can impede the induction of Th2-polarized immunity (91, 92), and patients infected with HTLV-1 have more frequent and more severe forms of strongyloidiasis. Patients co-infected with HTLV-1 and *S. stercoralis* were shown to have higher parasite burdens than those with strongyloidiasis alone (93). Mouse models have shown that IL-5-mediated eosinophil production and activation is important for protection against infection with *S. stercoralis* (94). In patients co-infected with HTLV-1 and *S. stercoralis* both parasite antigen-specific IL-5 responses and eosinophil levels were significantly decreased, suggesting an additional means by which the virus infection may impede immunity to helminths. However, rare incidences of acute respiratory distress syndrome have been encountered in HTLV-1-infected patients following treatment with antihelminthics (95). The mechanisms responsible for the development of this pathology are unknown, but it is plausible that acute immune reactions to the intrapulmonary destruction of the large parasite burden following antihelminthic treatment may play a role in triggering this response (95).

#### Vaccinia virus

A study in BALB/c mice has shown that co-infection with *Ascaris* in *Vaccinia* virus-infected hosts enhances the virus-associated pathology due to impaired *Vaccinia* virus-specific immunity (96). The levels of splenic CD8+ T cells in the co-infected mice were significantly reduced, as was the frequency of IFN-γ-producing virus-specific CD4^+^ and CD8^+^ T cells. Similar effects have also been reported in mice co-infected with *S. mansoni* (97). In this study *Vaccinia* virus-specific CD8^+^ cytotoxic T-cell responses were reduced in the mice co-infected with *S. mansoni*, suggesting a mechanistic link between the increased viral loads and reduced viral clearance. Since many chronic helminth infections induce a strong Th2-polarized immune response, the above examples suggest that the presence of this cytokine milieu at the time of virus co-infection may play an important role in impeding the induction of the IFNγ-mediated control of virus replication.

#### Respiratory syncytial virus (RSV)

In contrast to the above reports, it is plausible that in some circumstances that co-infection with helminths may enhance protection against viruses. Respiratory syncytial virus (RSV) is a major respiratory pathogen, and nearly all infants are infected with this virus by the age of 2 years old. However, because the virus does not induce lasting immunity recurrent RSV infections can occur throughout life. A study has shown how a gastrointestinal helminth infection can promote protective antiviral effects in the lung (98). Mice infected with *H. polygyrus* had reduced viral loads after co-infection with RSV and developed significantly less disease and pulmonary inflammation. These effects were not a considered to be a consequence of the induction of a Th2-polarized immune response to the worm infection. Instead, *H. polygyrus* infection coincided with the upregulated expression of type I IFN in the gut and the lung in a microbiota-dependent manner. Furthermore, the protective effects of helminth infection on RSV co-infection were impeded in mice lacking type I IFN receptor signaling (*Ifnar1*-deficient mice).

#### Virus infection in amphibians

All the above studies have described parasite virus co-infections in mammals or birds, but similar interactions have been reported in experimental amphibians. Prior infection of the larval stages (tadpole) of four distinct amphibian species with trematode parasites (*Echinoparyphium* spp.) significantly reduced viral loads following co-infection with ranavirus (99). Furthermore, *Echinoparyphium* co-infection coincided with reduced ranavirus transmission within a community of larval wood frogs (*Lithobates sylvaticus*). The cellular and molecular mechanisms by which helminth co-infection mediated these effects on ranavirus pathogenesis remain to be determined.

## Infections with prions (transmissible spongiform encephalopathies)

Prions are a unique group of pathogens that can cause infectious, chronic, neurodegenerative diseases in humans and some domesticated and free-ranging animal species. The precise nature of the infectious prion is uncertain, but an abnormal, relatively proteinase-resistant isoform (PrP^Sc^) of the host cellular prion protein (PrP^C^), co-purifies with prion infectivity in diseased tissues (100). Many natural prion diseases are acquired by oral consumption of contaminated food or pasture. The gut-associated lymphoid tissues (GALT) within the lining of the intestine such as the tonsils, Peyer's patches, appendix, colonic, and caecal patches, together with the mesenteric lymph nodes, help to provide protection against intestinal pathogens. However, the early replication of prions within the Peyer's patches in the small intestine is essential for their efficient spread of from the gut to the brain (a process termed *neuroinvasion*) (101–104).

Natural prion disease susceptible hosts such as sheep, deer, and cattle are regularly exposed to helminths but it is uncertain whether co-infections with these pathogens can influence oral prion disease pathogenesis, for example by causing damage to the gut epithelium and enhancing the uptake of prions into the GALT. In one study, lambs with high genetic susceptibility to natural sheep scrapie were experimentally co-infected with *Teladorsagia circumcincta* at monthly intervals from 6 to 11 months old and effects on prion disease determined (105). Although no mechanistic insights were reported, the authors suggested that the onset of prion disease was shortened in the co-infected lambs. However, the significance of data reported in this study is unclear as the animals were co-infected with *T. circumcincta* long after prion neuroinvasion from the intestine had occurred (106, 107). Conversely, when mice were co-infected with the large intestine-restricted helminth pathogen *T. muris* around the time of oral prion exposure, no effects on prion disease duration were observed (104). This is most likely because the large intestinal GALT are not important early sites of prion accumulation and neuroinvasion (104). Clearly additional studies are required to determine whether the pathology specifically in small intestine caused by a helminth infection may influence the prion neuroinvasion from the gut to the brain.

## Concluding remarks

Infectious diseases are commonly studied in experimental animals exposed to individual pathogenic microorganisms. However, this review described many examples of how infection with certain parasites can have a dramatic influence on host susceptibility or disease pathogenesis to co-infection with other pathogens. Many of these studies have reported correlations between alterations to specific immune parameters (T cell polarity etc.) and pathogen susceptibility, raising the hypothesis that many of the effects of co-infection are immune mediated. For example, alterations to the polarity of the T-cell response or macrophage phenotype induced by the parasite infection could affect the induction of protective immunity to co-infection with another pathogen. Further scrutiny of these studies shows that in many instances definitive demonstrations that the effects are indeed immune-mediated are lacking. Addressing these issues in natural host species is technically challenging. However, a large array of murine *in vivo* tractable systems are now available that enable the contributions of specific cellular and molecular immune components to be determined.

Laboratory mice housed to high microbiological status in specific-pathogen free conditions have proved to be highly tractable model systems in which to study the pathogenesis of many infectious diseases. How representative these mice are to natural host species in field conditions is questionable, since wild mice are typically infected with numerous micro- and macro-parasite species. The immune status of laboratory mice and wild mice differs significantly (108). Wild mice are markedly more antigen-experienced than laboratory mice, displaying on-going immune activation and the presence of an inflammatory myeloid cell subset that has not been detected in laboratory mice. Wild mice also express cytokine responses to microbial ligands that are similar or lower when compared to laboratory mice, and have highly heterogeneous gut microbiomes (108, 109). This suggests that the high level of pathogen exposure is a major driver of the enhanced immune activation in wild mice.

Many of the above examples suggest links between alterations to Th2/Th1 polarity or the nature of the innate immune response and susceptibility to pathogen co-infection. However, the underlying rules that dictate whether these interactions are likely to confer increased susceptibility or protection are not always apparent and are likely influenced by multiple factors. For example, susceptibility to these co-infections may be dependent on the individual niches that each pathogen inhabits in the host e.g., the same niche, or mucosal (gastrointestinal) vs. systemic. In other situations, the induction of a strong pro-inflammatory response to a parasite infection may exacerbate susceptibility and/or pathology following co-infection with another pathogen that induces a similar pro-inflammatory response. Chronic helminth infection in mice also promoted the reactivation of a latent virus infection. But not all the effects on co-infection appear to be directly immune mediated, as disruptions to the metabolic profile within the gastrointestinal tract following helminth infection promoted susceptibility to co-infection with certain pathogenic bacteria. As well as influencing host susceptibility to pathogen co-infection, the immune response to certain parasite infections may have other important health issues by negatively affecting the induction of antigen-specific immunity to vaccine antigens, reducing vaccine efficacy.

A greater understanding of how infectious disease susceptibility and pathogenesis are influenced by concurrent parasite infections will help the design of more effective treatments to control the spread of infectious diseases. For example, some helminth-derived ES products possess potent immunoregulatory properties, and these could be sufficient to suppress allograft rejection (110). Whether similar parasite-derived molecules can suppress host-responses to other pathogens, or conversely can be used therapeutically to enhance their clearance, remains to be determined.

## Author contributions

The author confirms being the sole contributor of this work and has approved it for publication.

### Conflict of interest statement

The author declares that the research was conducted in the absence of any commercial or financial relationships that could be construed as a potential conflict of interest.
